# Whole genome sequencing informs SNP-based breeding strategies to safeguard genetic diversity in captive African lions

**DOI:** 10.3389/fvets.2025.1577726

**Published:** 2025-04-16

**Authors:** Wanzhao Chen, Ronghe Xing, Panpan Xia, Yujie Yang, Chao Ma, Lining Xia

**Affiliations:** ^1^College of Veterinary Medicine, Xinjiang Agricultural University, Urumqi, China; ^2^Xinjiang Key Laboratory of New Drug Research and Development for Herbivores, Urumqi, China; ^3^Zoo Urumqi, Urumqi, China

**Keywords:** African lions, NGS, SNP, genetic diversity, captive breeding program

## Abstract

**Introduction:**

African lions (*Panthera leo*) face severe population declines, making captive breeding programs essential for conservation. However, genetic data scarcity in such programs elevates inbreeding risks and threatens genetic diversity.

**Methods:**

Using next-generation sequencing (NGS), we analyzed genome-wide genetic markers from 10 captive African lions at Xinjiang Tianshan Wildlife Park. We identified high-confidence SNPs, evaluated population structure, and calculated kinship/inbreeding coefficients alongside identity by descent (IBD) and identity by state (IBS) analyses.

**Results:**

We identified 5,051,795 high-confidence SNPs. The population contained distinct genetic subgroups. Six lion pairs exhibited elevated kinship coefficients, with one individual showing inbreeding signs. We developed a science-driven breeding program based on population genetic structure, identity by descent (IBD) analysis, and Identity by State (IBS) analysis. This program prioritizes pairings with low kinship while maintaining a balanced ancestral lineage.

**Discussion:**

This study underscores the importance of genomic tools in managing captive populations, offering actionable insights to mitigate inbreeding risks and improve long-term viability. This approach offers a model for optimizing breeding strategies in other endangered species conservation efforts.

## 1 Introduction

The African lion, the largest felid in Africa and the only sexually dimorphic big cat species globally, holds the iconic title of “King of the Savannah” (1, 2). African lions are now confined to <10% of their historical range, with surviving populations facing escalating human-lion conflicts, retaliatory killings due to livestock predation, and unsustainable trophy hunting pressures (2). Listed as Vulnerable on the IUCN Red List of Threatened Species, lion populations have declined by 30% over the past two decades (3). Ex situ conservation programs are critical for preserving genetic diversity, yet captive populations often suffer from founder effects and inbreeding (4, 5). Small populations experience accelerated genetic drift, leading to random reductions in heterozygosity (6). This increases the likelihood of fitness declines and the expression of deleterious recessive alleles, ultimately threatening population viability (7, 8). While microsatellite markers (short tandem repeats, STRs) have long served as the standard for assessing wildlife genetic diversity (9, 10), their limitations—including genotyping inaccuracies, homoplasy, and poor resolution for detecting fine-scale population structure—compromise their reliability in small or fragmented groups (11). Crucially, weak correlations between microsatellite-derived heterozygosity and true inbreeding coefficients may obscure extinction risks in managed populations (12).

This challenge is exemplified by Xinjiang Tianshan Wildlife Park’s captive breeding program, which imported 10 subadult African lions from South Africa without genetic or pedigree data. The absence of founder information raises concerns about unmanaged inbreeding over generations. To address this, we implemented NGS for genome-wide SNP genotyping. SNP markers overcome the limitations of microsatellites by enabling precise quantification of genetic diversity, high-resolution pedigree reconstruction, and detection of subtle kinship patterns through dense genome-wide coverage (11, 13, 14). For instance, SNP-based analyses in red deer revealed stronger heterozygosity-inbreeding correlations and finer population subdivisions than microsatellites, demonstrating their utility in conservation planning (11).

Importantly, the NGS-generated genomic data from this study contribute to expanding lion genome databases and enable comparative analyses between wild and captive populations. Such datasets are critical for refining phylogenetic frameworks, identifying adaptive loci, and informing translocations to enhance metapopulation resilience.

By employing NGS on the 10 captive African lions, we systematically assessed genetic diversity, elucidated population structure, and constructed high-resolution pedigrees. These results provide actionable insights for science-driven breeding programs while contributing foundational genomic data to global lion conservation frameworks.

## 2 Materials and methods

### 2.1 Sample collection and DNA extraction

In this study, 10 subadult African lions bred in captivity at Xinjiang Tianshan Wildlife Park were systematically numbered TSFZS1-TSFZS10 with biological profiles as follows: TSFZS1 (♀, 2 yr), TSFZS2 (♀, 2 yr), TSFZS3 (♀, 3 yr), TSFZS4 (♂, 3 yr), TSFZS5 (♀, 2 yr), TSFZS6 (♂, 2 yr), TSFZS7 (♂, 3 yr), TSFZS8 (♀, 2 yr), TSFZS9 (♀, 3 yr), and TSFZS10 (♀, 2 yr). With the help of a zoo veterinarian, we collected whole blood (approximately 2.0 mL) from each individual’s jugular vein using a blood collection needle. We then placed it in a blood collection tube containing EDTA.

DNA from the samples sequenced in this study was extracted using the DNeasy Blood & Tissue kit (Qiagen, Hilden, Germany). The integrity, purity, and concentration of the DNA were assessed using the Fragment Analyzer 5,400 (Agilent Technologies, Inc., Beijing, China).

### 2.2 Library preparation and Illumina sequencing

Tianjin Novogene Bioinformatics Technology Co., Ltd. performed library preparation and sequencing. Qualified DNA samples were randomly sheared into fragments of approximately 350 bp using a Covaris S2 sonicator (Covaris, Woburn, Massachusetts, United States). The processed DNA fragments were then subjected to library construction using the NEBNext^®^ Ultra™ II DNA Library Prep Kit for Illumina^®^ (New England Biolabs, United States), following standard procedures including end repair, A-tailing, adapter ligation, purification, and PCR amplification.

Following library preparation, initial quantification was conducted using a Qubit^®^ 2.0 Fluorometer (Life Technologies, CA, United States), and libraries were diluted to 1 ng/μl. The insert size distribution was subsequently analyzed using the Fragment Analyzer 5,400 system. Upon confirmation of the expected insert sizes, q-PCR was employed to precisely determine the effective library concentration (>2 nM), ensuring final library quality. After passing library quality control, Illumina PE150 paired-end sequencing was conducted based on the effective library concentration and required data output specifications.

### 2.3 Sequencing data quality control and bioinformatics analysis

#### 2.3.1 Sequencing data quality control

Raw image data generated from PE150 paired-end sequencing were converted into sequence data (raw data) by base calling. Raw data were filtered using FastQC with the following criteria: remove read pairs containing adapter sequences; discard paired reads if ≥ 10% of bases in either single-end read are ambiguous (N bases); discard paired reads if >50% of bases in either single-end read have low-quality scores (Q ≤ 5) (15).

High-quality clean data were obtained after filtration, and sequencing output metrics were calculated. Clean data were aligned to the lion reference genome^1^ using BWA (Version: 0.7.8-r455) (16). Aligned reads were sorted and PCR duplicates were removed using SAMtools (Version: 1.3.1) (17).

#### 2.3.2 SNP calling

Population SNPs were identified using SAMtools (Version: 1.3.1)/BCFtools (Version: 1.4) (18) pipeline with the following filtering thresholds: DP: Minimum read depth per sample per SNP = 3 (sites below this depth marked as missing); MISS: Maximum missing rate per SNP = 0.1 (SNPs exceeding this threshold excluded); MAF: Minimum minor allele frequency = 0.05 (SNPs below this frequency excluded). Final SNPs were annotated using ANNOVAR (19) for functional characterization.

#### 2.3.3 Population genetic structure analysis

Principal Component Analysis (PCA): Performed on filtered SNPs using GCTA (Version: 1.24.2) (20) to generate sample-specific scores for the first three principal components (PC1–PC3), and visualized via scatterplots.

Phylogenetic Tree Construction: A pairwise distance matrix was computed using TreeBest (Version: 1.24.2)^2^, followed by neighbor-joining tree reconstruction.

Genetic Structure Inference: Ancestry proportions and population lineages were estimated using ADMIXTURE (Version: 1.23) (21).

#### 2.3.4 Identity by descent

PLINK (Version: 1.9) (22) was employed to calculate Z-scores for IBD segments, determining kinship coefficients between samples.

#### 2.3.5 Identity by stat

Kinship coefficients between individuals were calculated using GCTA (Version: 1.24.2) based on the allele-sharing similarity between individuals.

#### 2.3.6 Inbreeding coefficient

PLINK (Version: 1.9) was used to estimate the inbreeding coefficient (F) for each sample, quantifying the probability of shared ancestral homozygosity.

### 2.4 Formulating a captive breeding program

The proposed pairings prioritize individuals with low kinship coefficients (PI_HAT <0.125) and complementary ancestral lineages identified through PCA and ADMIXTURE (Version: 1.23) analyses.

## 3 Results

### 3.1 Sequencing data quality assessment

Statistical results demonstrated that the 10 samples generated an average of 31.90 Gb of raw data per individual (total raw data: 319.03 Gb), with 31.60 Gb of clean data per individual retained after filtering (total clean data: 316.03 Gb). Sequencing quality metrics included Q20 ≥ 97.14%, Q30 ≥ 92.50%, and GC content ranging from 41.29 to 42.66% across samples (Supplementary Table S1). The raw reads used in this article have been deposited into the Sequence Read Archive (SRA) of the NCBI database under BioProject accession number: PRJNA1222837.

### 3.2 Reference genome alignment

The lion reference genome (2,297,568,983 bp; Supplementary Table S2) demonstrated alignment rates of 96.26–98.03% across all samples, with post-alignment metrics including coverage depth (≥ 4×, excluding N-regions) of 8.98 × −11.02×, 1 × coverage (≥ 1 bp aligned) of 99.04–99.78%, and 4 × coverage (≥ 4 bp aligned) of 93.15–96.68% (Supplementary Table S3).

### 3.3 SNP calling

A total of 6,010,278 qualified SNPs were initially identified in the sampled population. After stringent quality control and filtering, 5,051,795 high-confidence SNPs were retained for downstream analyses. Functional annotations of these SNPs are summarized in Supplementary Table S3.

### 3.4 Population genetic structure analysis

**Figure 1:**
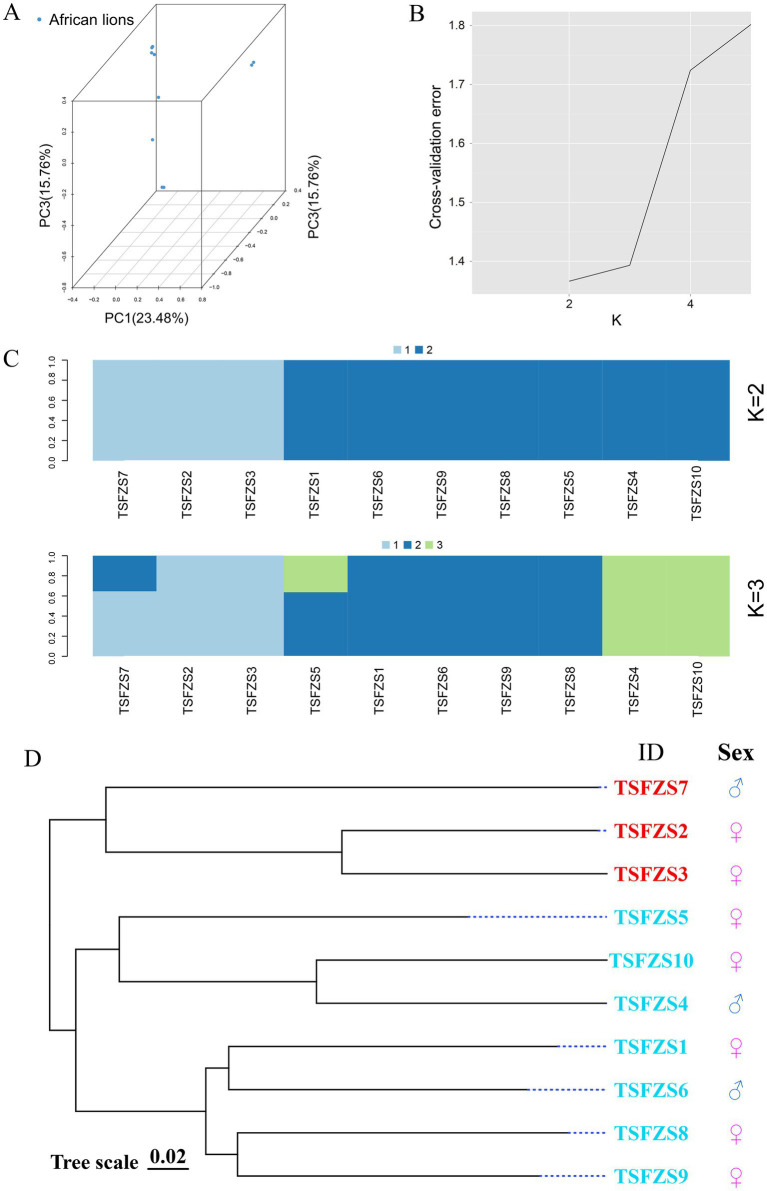
Population genetic structure analysis. **(A)** Three-dimensional PCA of lion genomic SNPs. The X, Y, and Z-axis coordinates represent principal component 1 (PC1), principal component 2 (PC2), and principal component 3 (PC3), respectively. Each point in the plot corresponds to an individual sample. The clustering pattern of samples reflects their genetic relatedness, with outliers potentially indicating divergence from the main population; **(B)** Optimal *K*-value selection; **(C)** Population structure clustering (*K* = 2–4). The horizontal axis represents individual identifiers, while the vertical axis indicates the proportion of ancestral genetic components. The annotations on the left (*K* = 2 to *K* = 4) indicate the hypothesized range of ancestral population numbers inferred in this study, spanning from 2 to 4 genetically distinct groups; **(D)** Phylogenetic tree. The phylogenetic tree topology visually illustrates the evolutionary relationships among different populations, with closely related varieties clustering together in the tree.

Principal component analysis (PCA) was performed using GCTA software, with results visualized in Figure 1A. Based on PC1, PC2, and PC3, the 10 lion samples were clustered into five subgroups: Subgroup 1 comprised 4 individuals, Subgroups 2 and 3 each contained 2 individuals, and Subgroups 4 and 5 each included 1 individual.

Population genetic structure analysis using ADMIXTURE revealed the optimal number of ancestral populations (*K*) through cross-validation (CV) error minimization (Figure 1B). Following the ADMIXTURE manual guidelines, *K* = 2 was identified as the optimal value. At *K* = 2, the population diverged into two distinct subgroups with 3 and 7 individuals, respectively (Figure 1C). When *K* = 3, the cohort separated into five subgroups, consistent with PCA results. Further subdivision at *K* ≥ 4 yielded biologically insignificant clusters due to deviation from the optimal *K* value.

Phylogenetic analysis (Figure 1D) resolved the 10 samples into two major clades: one comprising 7 individuals and the other containing 3 individuals, aligning with the *K* = 2 genetic structure results.

### 3.5 IBD analysis

Pairwise kinship analysis of the 10 African lions revealed substantial genetic relatedness, with inbreeding coefficients (PI_HAT) values ranging from 0 to 0.543 (Table 1). Among the 10 African lions, 15 pairwise comparisons with PI_HAT values exceeding 0.125.

**Table 1:** Pairwise kinship coefficients among lion samples.

IID1	IID2	Z0	Z1	Z2	PI_HAT
TSFZS2	TSFZS3	0.216	0.483	0.302	0.543
TSFZS4	TSFZS10	0.298	0.456	0.247	0.475
TSFZS6	TSFZS9	0.265	0.574	0.161	0.448
TSFZS1	TSFZS6	0.316	0.489	0.196	0.440
TSFZS8	TSFZS9	0.333	0.470	0.198	0.433
TSFZS6	TSFZS8	0.311	0.553	0.136	0.412
TSFZS1	TSFZS9	0.381	0.487	0.132	0.376
TSFZS1	TSFZS8	0.472	0.438	0.090	0.309
TSFZS5	TSFZS9	0.501	0.499	0.000	0.249
TSFZS5	TSFZS8	0.535	0.465	0.000	0.232
TSFZS5	TSFZS6	0.536	0.464	0.000	0.232
TSFZS1	TSFZS5	0.567	0.434	0.000	0.217
TSFZS6	TSFZS10	0.637	0.363	0.000	0.181
TSFZS4	TSFZS5	0.849	0.000	0.151	0.151
TSFZS9	TSFZS10	0.727	0.273	0.000	0.137
TSFZS5	TSFZS10	0.842	0.065	0.093	0.126
TSFZS1	TSFZS10	0.792	0.208	0.000	0.104
TSFZS4	TSFZS6	0.817	0.183	0.000	0.092
TSFZS1	TSFZS4	0.864	0.136	0.000	0.068
TSFZS8	TSFZS10	0.866	0.134	0.000	0.067
TSFZS4	TSFZS9	0.879	0.121	0.000	0.061
TSFZS7	TSFZS9	1.000	0	0	0
…	…	…	…	…	…
TSFZS1	TSFZS2	1.000	0	0	0

IID1: sample 1; IID2: sample 2; Z0 represents the probability that samples 1 and 2 share zero chromosomal regions with identical variants. Similarly, Z1 and Z2 denote the probabilities of the two samples sharing one or two chromosomal regions, respectively, with identical variants; PI_HAT: IBD proportion. The relationship between PI_HAT values and kinship categories is as follows: PI_HAT = 0: Unrelated individuals; PI_HAT = 0.25: First cousins; PI_HAT = 0.5: Parent-offspring or full siblings; PI_HAT = 1: Same individual or monozygotic twins.

### 3.6 G-matrix analysis of kinship coefficients

**Figure 2:**
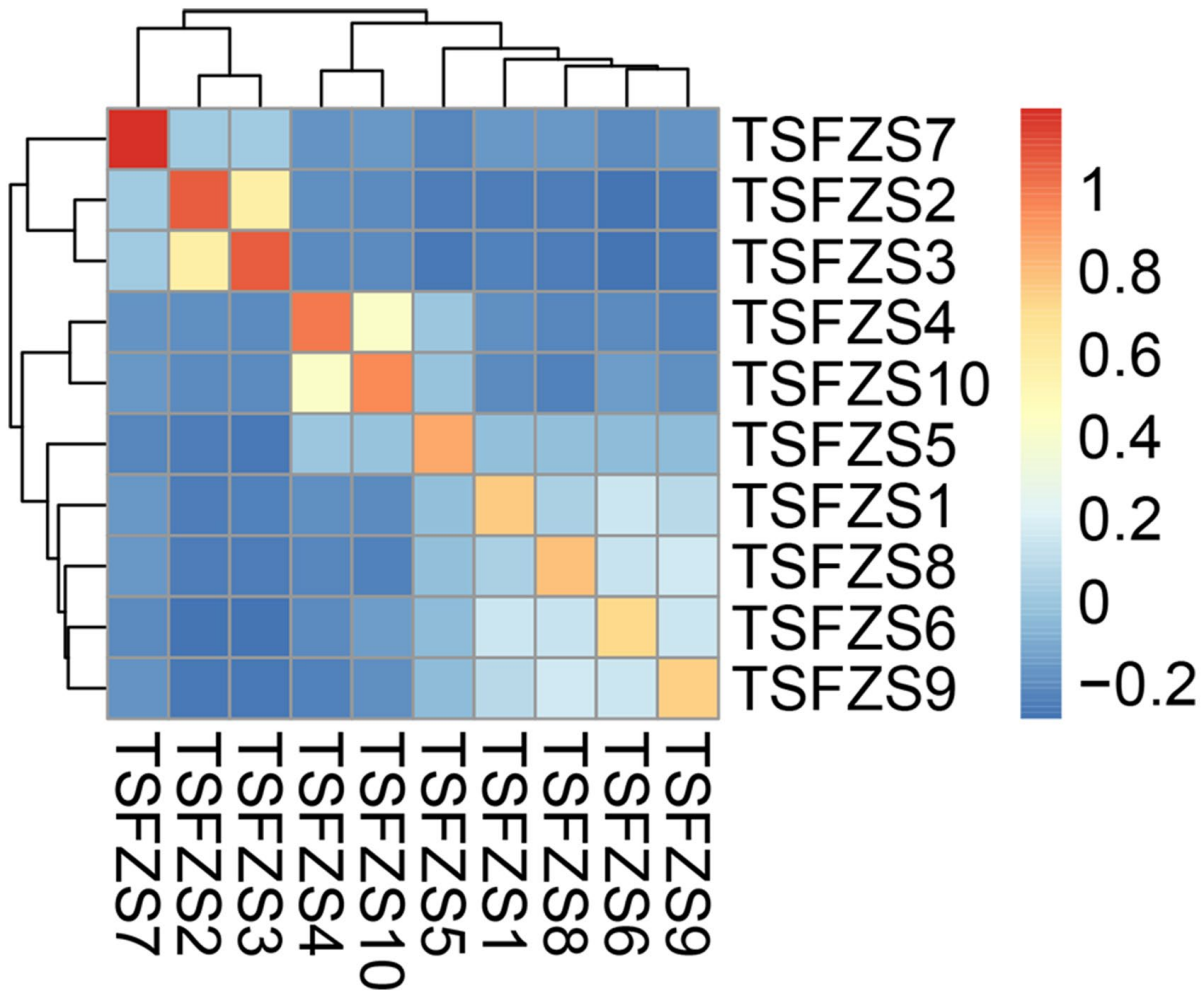
Pairwise kinship coefficient among the 10 captive African lions. The heatmap represents the pairwise kinship coefficient values between individuals in the 10 captive African lions. The color scale ranges from blue (low kinship coefficient) to red (high kinship coefficient), with white indicating intermediate values. The dendrograms on the top and left sides of the heatmap display the hierarchical clustering of individuals based on their kinship coefficient patterns.

The kinship coefficient matrix (Figure 2; Supplementary Table S4) revealed diverse genetic kinship coefficient among 10 captive African lions, ranging from −0.285 to 1.194. Self-kinship coefficient values (diagonal entries) were consistently high and negative kinship coefficients predominantly dominated pairwise comparisons. However, there were six pairs of combinations with average kinship coefficient values greater than 0.125, specifically: TSFZS2-TSFZS3 (0.582); TSFZS4-TSFZS10 (0.412); TSFZS1-TSFZS6 (0.149); TSFZS8-TSFZS9 (0.164); TSFZS6-TSFZS9 (0.148); TSFZS6-TSFZS8 (0.130).

### 3.7 Inbreeding coefficient

Genetic analysis of 10 captive African lions revealed substantial heterogeneity of inbreeding coefficients (*F*) across individuals (Table 2). Nine individuals exhibited negative *F* values (range: −0.1828 to −0.0317). Notably, one individual (TSFZ55) displayed a positive *F* value (0.1375). Observed genotype counts (OBS_CT) ranged consistently between 4,922,689 and 4,998,117, confirming high data coverage and analytical robustness.

**Table 2:** Inbreeding coefficient value.

IID	O(HOM)	E(HOM)	OBS_CT	*F*
TSFZS1	3,129,510	3.345e+06	4,946,883	−0.1345
TSFZS2	3,314,884	3.366e+06	4,979,434	−0.03171
TSFZS3	3,266,365	3.368e+06	4,982,253	−0.06297
TSFZS4	3,273,628	3.328e+06	4,922,689	−0.03421
TSFZS5	3,557,745	3.338e+06	4,935,916	0.1375
TSFZS6	3,088,445	3.379e+06	4,998,117	−0.1798
TSFZS7	3,236,308	3.349e+06	4,953,761	−0.07032
TSFZS8	3,089,595	3.351e+06	4,955,482	−0.1628
TSFZS9	3,069,655	3.364e+06	4,975,443	−0.1828
TSFZS10	3,159,623	3.368e+06	4,981,509	−0.1291

O(HOM): Number of observed homozygous genotypes; E(HOM): Number of expected homozygous genotypes; OBS_CT: Total number of observed genotypes; F: Inbreeding coefficient.

### 3.8 Captive breeding program

**Figure 3:**
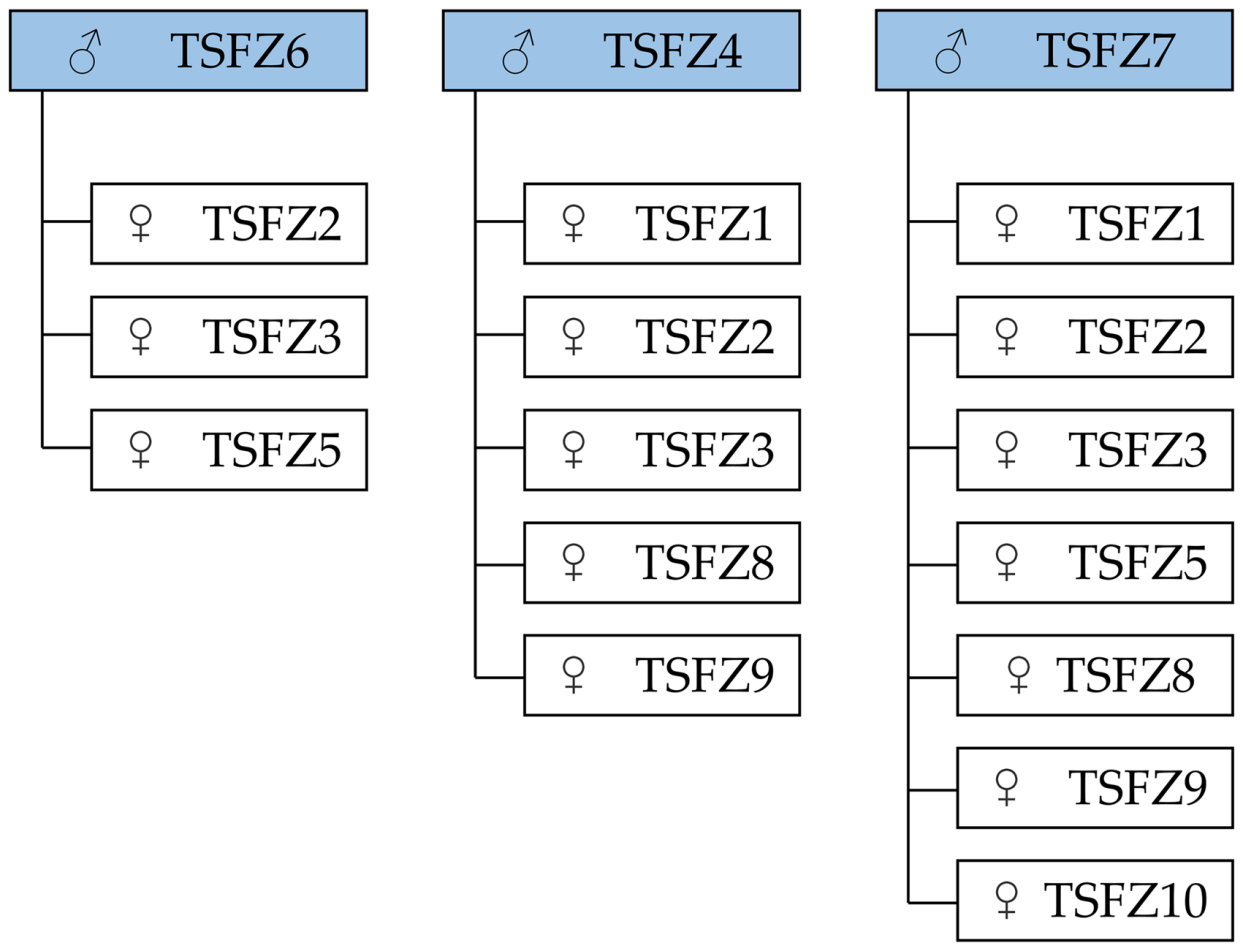
Mating plan of pairs suitable for reproduction.

Based on population genetic structure, IBD analysis, and IBS analysis, a targeted breeding plan was established to minimize inbreeding risks (Figure 3). Male lion TSFZS6 was paired with female lions TSFZS2, TSFZS3, and TSFZS5. Male lion TSFZS4 was assigned to females TSFZS1, TSFZS2, TSFZS3, TSFZS8, and TSFZS. Male lion TSFZS7 exhibited broad compatibility, with recommended pairings including seven females (TSFZS1, TSFZS2, TSFZS3, TSFZS5, TSFZS6, TSFZS9, TSFZS10).

## 4 Discussion

This study assessed the genetic diversity and kinship structure of a captive African lion population at Xinjiang Tianshan Wildlife Park using NGS. Our findings provide insights into the genetic health of the population and highlight the importance of informed breeding management to mitigate inbreeding risks. Genome-wide SNP genotyping demonstrated superior precision in genetic diversity assessment compared to traditional microsatellite analyses (23–25), aligning with global advancements in wildlife conservation. For instance, NGS-based metabarcoding has enabled non-invasive dietary profiling of endangered Asian elephants (*Elephas maximus*) in Southeast Asia (26), while whole-genome sequencing of African lions has resolved critical phylogeographic divisions (e.g., northern vs. southern subspecies divergence) and facilitated targeted SNP panels for anti-poaching forensics and ex situ breeding programs (27). Collectively, these studies highlight NGS’s pivotal role in diagnosing genetic vitality and guiding conservation strategies across ecosystems.

PCA and ADMIXTURE clustering revealed distinct genetic subgroups within the population. While the optimal ancestral population number (*K* = 2) aligns with Tanzania’s wild African lion populations, the finer-scale clustering (*K* = 3) reflects localized founder effects and genetic drift, which are also observed in fragmented wild populations (28). Analysis of kinship coefficients revealed substantial variation in relatedness, with several individuals showing elevated inbreeding coefficients (PI_HAT >0.125). Similar patterns have been observed in other captive populations, where limited founder numbers and unregulated mating may lead to elevated inbreeding coefficients and declining genetic viability (23–25, 29). The dominance of negative kinship coefficients is in contrast to wild lion populations, where positive relatedness is common due to philopatry (30).

Our study reveals a genetically healthy captive lion population, with negative inbreeding coefficients (F) in 9/10 individuals, indicating heterozygosity excess relative to Hardy–Weinberg expectations (31). However, the outlier TSFZS5 (*F* = 0.1375) suggests increased homozygosity, possibly due to undocumented consanguineous mating or skewed founder contributions. Long-term monitoring of TSFZS5’s lineage is critical to assessing the fitness impacts of elevated homozygosity.

The captive breeding program formulated in this study aims to suppress genetic drift and enhance long-term evolutionary potential of the population by avoiding high-kinship pairings (kinship coefficient >0.125) and establishing inter-subpopulation gene flow mechanisms (32). Subsequent program iterations will systematically integrate genomic monitoring frameworks to periodically evaluate breeding outcomes and optimize mating strategies, ensuring technical protocols maintain compliance with IUCN Species Survival Commission (SSC) ex situ conservation standards (33). This study’s small sample size (*n* = 10) may limit the detection of rare alleles and fine-scale substructure, yet the identification of distinct genetic subgroups and high kinship pairs underscores the utility of genomic tools in guiding captive breeding. Despite potential constraints, the proposed framework aligns with conservation strategies for endangered species, where actionable insights emerge even from limited datasets (34, 35). Expanding genomic monitoring to larger cohorts (e.g., 120 Przewalski’s horses at Xinjiang Tianshan Wildlife Park) and integrating wild population data will enhance the model’s applicability across fragmented or reintroduced populations.

## 5 Conclusion

This study used NGS to assess the genetic diversity and kinship of captive African lions at Xinjiang Tianshan Wildlife Park. Our findings revealed significant genetic variation and distinct subgroups within the population. High kinship coefficients in several pairs of individuals highlighted the risks of inbreeding and genetic drift. Based on these genomic insights, we developed a breeding program prioritizing low-kinship pairings to minimize inbreeding risks and maintain genetic diversity, which is vital for the population’s long-term survival. These results demonstrate the power of genomic tools in shaping effective breeding strategies for conservation. Future studies should expand the genomic dataset to include additional captive and wild populations to further refine conservation strategies. This approach provides valuable insights for managing other endangered species in captive settings and contributes to global lion conservation efforts.

## Data Availability

The datasets presented in this study can be found in online repositories. The names of the repository/repositories and accession number(s) can be found in the article/Supplementary material.

## Footnotes

^1^ https://ftp.ncbi.nlm.nih.gov/gnomes/all/GCF/018/350/215/GCF_018350215.1_P.leo_Ple1_pat1.1/GCF_018350215.1_P.leo_Ple1_pat1.1_genomic.gff

^2^ http://treesoft.sourceforge.net/treebest.shtml
