# Supplementary material for: Whole genome sequencing informs SNP-based breeding strategies to safeguard genetic diversity in captive African lions
Source: Front Vet Sci. 2025 Apr 16;12:1577726. doi: 10.3389/fvets.2025.1577726 (PMC12040825; doi:10.3389/fvets.2025.1577726)
Supplement: Supplementary file 1 [file Table_1.docx]

Supplementary Material

**Table S1****.** Sequencing Data Quality Assessment

| sample | Raw bases (bp) | Clean bases (bp) | Effective rate (%) | Error rate(%) | Q20(%) | Q30(%) | GC content(%) |
| --- | --- | --- | --- | --- | --- | --- | --- |
| TSFZS1 | 29552776200 | 29289715500 | 99.11 | 0.01 | 97.27 | 92.88 | 41.47 |
| TSFZS10 | 33843485100 | 33555658500 | 99.15 | 0.01 | 97.33 | 93.02 | 41.29 |
| TSFZS2 | 33286569600 | 32915526000 | 98.89 | 0.01 | 97.14 | 92.50 | 41.31 |
| TSFZS3 | 32207208600 | 31924448400 | 99.12 | 0.01 | 97.38 | 93.19 | 41.55 |
| TSFZS4 | 31798847100 | 31497384300 | 99.05 | 0.01 | 97.40 | 93.29 | 42.66 |
| TSFZS5 | 29853574200 | 29567292600 | 99.04 | 0.01 | 97.23 | 92.69 | 41.40 |
| TSFZS6 | 34816866900 | 34459311000 | 98.97 | 0.01 | 97.41 | 93.28 | 42.23 |
| TSFZS7 | 30283145700 | 30000243600 | 99.07 | 0.01 | 97.33 | 93.03 | 41.58 |
| TSFZS8 | 30780555300 | 30510183600 | 99.12 | 0.01 | 97.19 | 92.65 | 41.50 |
| TSFZS9 | 32602474500 | 32312844000 | 99.11 | 0.01 | 97.18 | 92.52 | 41.60 |

Note: Raw bases (bp): Total raw sequencing output per sample in base pairs; Clean bases (bp): Post-QC filtered sequencing data in base pairs; Effective rate (%): Ratio of clean bases to raw bases (Clean bases / Raw bases × 100); Error rate (%): Mean base-calling error probability; Q20 (%): Proportion of bases with Phred quality score ≥20 (error rate ≤1%); Q30 (%): Proportion of bases with Phred quality score ≥30 (error rate ≤0.1%); GC content (%): Percentage of guanine (G) and cytosine (C) bases in the sequenced data.

**Table S2. Reference Genome Information**

| Seq num | Total length | GC content(%) | Gap rate(%) | N50 length | N90 length |
| --- | --- | --- | --- | --- | --- |
| 54 | 2,297,568,983 | 41.71% | 0.0004% | 147,402,474 | 69,419,297 |

Note: Seq num: Total number of sequences in the genome assembly; Total length: Cumulative length of the assembled genome; GC content (%): Percentage of guanine (G) and cytosine (C) bases; Gap rate (%): Proportion of unresolved regions (denoted as "N") in the assembly; N50 length: Scaffold N50 value, representing the minimum scaffold length at which 50% of the total assembly length is contained in scaffolds of this size or larger; N90 length: Scaffold N90 value, indicating the minimum scaffold length covering 90% of the total assembly.

**Table S3. Sequencing depth and coverage statistics**

| Sample | Clean_reads | mapped_reads | mapping_rate | Average_depth(4X) | Coverage_1X | Coverage_4X |
| --- | --- | --- | --- | --- | --- | --- |
| TSFZS9 | 215418960 | 207361982 | 96.26 | 9.59 | 99.56 | 96.17 |
| TSFZS2 | 219436840 | 211466743 | 96.37 | 9.91 | 99.57 | 96.67 |
| TSFZS10 | 223704390 | 215632630 | 96.39 | 9.92 | 99.56 | 96.68 |
| TSFZS3 | 212829656 | 205220297 | 96.42 | 10.07 | 99.54 | 96.63 |
| TSFZS1 | 195264770 | 188293771 | 96.43 | 9.18 | 99.52 | 95.20 |
| TSFZS5 | 197115284 | 190127123 | 96.45 | 8.98 | 99.49 | 94.64 |
| TSFZS8 | 203401224 | 196182920 | 96.45 | 9.27 | 99.53 | 95.47 |
| TSFZS4 | 209982562 | 205731469 | 97.98 | 10.35 | 99.04 | 93.15 |
| TSFZS6 | 229728740 | 225207575 | 98.03 | 11.02 | 99.60 | 95.97 |
| TSFZS7 | 200001624 | 196065897 | 98.03 | 9.17 | 99.78 | 95.61 |

Notes: mapped_reads: Number of clean reads successfully aligned to the reference genome; mapping_rate: Percentage of clean reads aligned to the reference genome relative to the total clean reads; average_depth(4X): Mean sequencing depth calculated as the total aligned bases in regions with ≥4× coverage divided by the size of the 4×-covered genomic regions; coverage_1X: Percentage of the reference genome covered by at least 1× read depth; coverage_4X: Percentage of the reference genome covered by at least 4× read depth.

**Table S4. SNP annotation results statistics**

| If_exonic | Category | Number of SNPs |
| --- | --- | --- |
|  | Upstream | 30838 |
|  | UTR3 | 29935 |
|  | UTR5 | 12937 |
|  | UTR5;UTR3 | 25 |
| Exonic | Stop gain | 176 |
| Exonic | Stop loss | 22 |
| Exonic | Synonymous | 23782 |
| Exonic | Non-synonymous | 17578 |
| Exonic | unknown | 998 |
|  | Intronic | 1968176 |
|  | Splicing | 157 |
|  | Downstream | 34937 |
|  | upstream/downstream | 1063 |
|  | Intergenic | 2931097 |
|  | Other | 74 |
|  | ts | 3560103 |
|  | tv | 1491692 |
|  | ts/tv | 2.386 |
|  | Total | 5051795 |

Notes: If_exonic: whether it is in an exon; the second column; Category: category; Based on the position of SNPs within gene structures, they can be categorized into the following types: Upstream: region 1 Kb upstream of the gene; 3’ UTR: 3’ untranslated region; 5’ UTR: 5’ untranslated region; 5’ UTR/3’ UTR: located in the 5’ UTR region of one gene and also in the 3’ UTR region of another gene; Exonic Non-synonymous: non-synonymous variation located in an exon, where the base change at the SNP site alters the type of amino acid encoded; Exonic synonymous: synonymous variation located in an exon, where the base change at the SNP site does not alter the type of amino acid encoded; Exonic stop gain: variation located in an exon that introduces a stop codon; Exonic stop loss: variation located in an exon that causes the loss of a stop codon; Exonic unknown: variation located in an exon that is not one of the above four types and whose significance is unknown; Intronic: variation located in an intron region; Splicing: variation located at splice sites (within 2 bp of the exon/intron boundary); Downstream: region 1 Kb downstream of the gene; Upstream/Downstream: region 1 Kb upstream of the gene and also in the downstream region 1 Kb of another gene; Intergenic: variation located in the intergenic region; Other: other types of variations; Based on the type of base substitution, they can be divided into transitions (ts) and transversions (tv): ts: transition, replacement between bases of the same type, such as between purines or between pyrimidines; tv: transversion, replacement between bases of different types, i.e., conversion between purines and pyrimidines; ts/tv: the ratio of the number of transition SNPs to the number of transversion SNPs; Total: total number of SNPs.

**Table S5. Kinship coefficient matrix**

| #IBS | TSFZS1 | TSFZS2 | TSFZS3 | TSFZS4 | TSFZS5 | TSFZS6 | TSFZS7 | TSFZS8 | TSFZS9 | TSFZS10 |
| --- | --- | --- | --- | --- | --- | --- | --- | --- | --- | --- |
| TSFZS1 | 0.7636 | -0.2451 | -0.2402 | -0.1854 | -0.0312 | 0.1489 | -0.1646 | 0.0439 | 0.0942 | -0.1986 |
| TSFZS2 | -0.2451 | 1.0643 | 0.5816 | -0.1918 | -0.2466 | -0.2851 | 0.0217 | -0.2498 | -0.2646 | -0.2068 |
| TSFZS3 | -0.2402 | 0.5816 | 1.0751 | -0.2085 | -0.2632 | -0.2708 | 0.0159 | -0.2504 | -0.2578 | -0.2043 |
| TSFZS4 | -0.1854 | -0.1918 | -0.2085 | 0.9943 | 0.0007 | -0.1980 | -0.1810 | -0.2217 | -0.2386 | 0.4123 |
| TSFZS5 | -0.0312 | -0.2466 | -0.2632 | 0.0007 | 0.8615 | -0.0476 | -0.2124 | -0.0201 | -0.0375 | -0.0174 |
| TSFZS6 | 0.1489 | -0.2851 | -0.2708 | -0.1980 | -0.0476 | 0.7183 | -0.2061 | 0.1303 | 0.1483 | -0.1504 |
| TSFZS7 | -0.1646 | 0.0217 | 0.0159 | -0.1810 | -0.2124 | -0.2061 | 1.1941 | -0.1577 | -0.1707 | -0.1621 |
| TSFZS8 | 0.0439 | -0.2498 | -0.2504 | -0.2217 | -0.0201 | 0.1303 | -0.1577 | 0.7841 | 0.1639 | -0.2376 |
| TSFZS9 | 0.0942 | -0.2646 | -0.2578 | -0.2386 | -0.0375 | 0.1483 | -0.1707 | 0.1639 | 0.7438 | -0.1951 |
| TSFZS10 | -0.1986 | -0.2068 | -0.2043 | 0.4123 | -0.0174 | -0.1504 | -0.1621 | -0.2376 | -0.1951 | 0.9477 |

Notes: Each cell represents the kinship coefficient between the sample on the x-axis and the corresponding sample on the y-axis of Figure 2.
